# Polydopamine Derived NaTi_2_(PO_4_)_3_–Carbon Core–Shell Nanostructures for Aqueous Batteries and Deionization Cells

**DOI:** 10.1021/acsanm.3c01687

**Published:** 2023-06-14

**Authors:** Nadežda Traškina, Gintarė Gečė, Jurgis Pilipavičius, Linas Vilčiauskas

**Affiliations:** † Center for Physical Sciences and Technology (FTMC), Saulėtekio al. 3, Vilnius LT-10257, Lithuania; ‡ Insitute of Chemistry, Vilnius University, Saulėtekio al. 3, Vilnius LT-10257, Lithuania

**Keywords:** sodium titanium phosphate, NASICON, aqueous sodium-ion batteries, carbon coating control, core−shell carbon layer

## Abstract

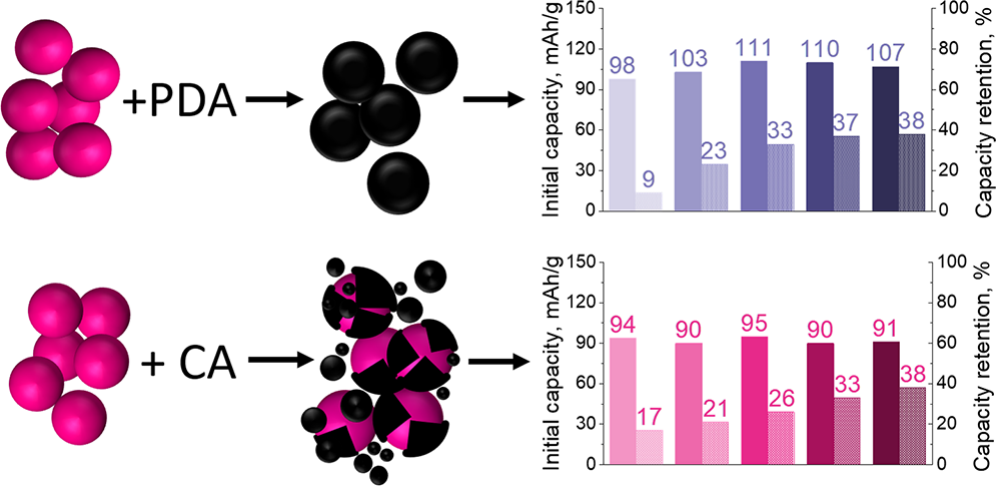

Due to their stability and structural freedom, NASICON-structured materials such as NaTi_2_(PO_4_)_3_ show a lot of promise as active electrode materials for aqueous batteries and deionization cells. However, due to their low intrinsic electronic conductivity, they must usually be composited with carbon to form suitable electrodes for power applications. In this work, two series of NaTi_2_(PO_4_)_3_–carbon composite structures were successfully prepared by different approaches: postsynthetic pyrolytic treatment of citric acid and surface polymerized dopamine. The latter route allows for a superior carbon loading control and yields more uniform and continuous particle coatings. The homogeneity of the polydopamine derived core–shell carbon layer is supported by FTIR, TEM, and XPS analysis. Combustion elemental analysis also indicates significant nitrogen doping in the final carbonaceous structure. The galvanostatic charge and discharge cycling results show similar initial capacities and their retention, but at only half of the carbon loading in polydopamine derived samples. The overall results indicate that careful nanostructure engineering could yield materials with superior properties and stability suitable for various electrochemical applications such as aqueous Na-ion batteries and deionization cells.

## Introduction

Sodium ion batteries have recently emerged as a viable alternative to lithium-ion batteries in the field of stationary large-scale energy storage applications.^1^ NASICON-structured materials show a lot of promise due to their high ionic conductivity, structural stability, and ability to tune the electrode potentials by the appropriate selection of transition metals.^2^ In addition to these advantages, there are also some drawbacks such as very low intrinsic electronic conductivity and limited stability in some electrolytes.^3^ NASICON-structured NaTi_2_(PO_4_)_3_ (NTP), with a potential of ca. −0.6 V (vs SHE) and a theoretical specific capacity of 133 mAh/g, is one of the most popular and widely studied negative electrode materials for various aqueous electrochemical devices such as Na-ion batteries or Faradaic deionization cells.^3−7^ The improvement of NTP electronic conductivity is very important for the applications of this material in real electrochemical devices where high currents must be sustained for extended periods of time.^8^ Some attempts to enhance the electronic conductivity focused on reducing the particle size, aliovalent doping, and particle coating with various conductive materials.^9−11^

In this work we study carbonaceous particle coatings that form a protective but electrically conductive layer allowing the passage of ions and electrons.^12−14^ Currently, there are several widely utilized approaches for forming such layers on ceramic materials. For example, one is based on simple mechanical dispersion and mixing of powder with a carbonaceous additive. Another class of methods uses the pyrolysis of carbon precursors introduced during synthesis or postsynthetic treatment in inert atmospheres.^15,16^ Because most forms of carbonaceous materials are neither soluble nor easily dispersible in traditional solvents, the latter approach provides a more uniform distribution and deposition of carbonaceous species typically resulting in better conductivities and charge capacities.^17^ However, the pyrolysis based methods do not typically result in very uniform particle coatings because some carbon inevitably remains intraparticular during the formation process.^14,18^ Although some of these issues might be mitigated by additional grinding and homogenization procedures, such coatings are still relatively irregular and uneven in terms of particle coverage.

A completely different approach is based on selective precursor adsorption and controlled polymerization in a thin shell on a particle surface. This method typically yields a much more uniform material encapsulation by carbon shells.^19−21^ Dopamine (DA) is a natural compound with superior adhesion to virtually any surface originating from multiple catechol and amine groups.^14,22^ The self-polymerization process of DA in a weakly basic medium under aerobic conditions produces a uniform tightly packed polydopamine (PDA) film.^23^ The subsequent pyrolysis of PDA, results in a graphitization of the polymerized shell yielding a thin and uniform carbon layer on the surface of a ceramic particle.^24^ In this study, we use a surface polymerization of DA to form a uniform PDA coating on NTP particles, which is eventually pyrolyzed in nitrogen atmosphere at >700 °C.

**Figure 1:**
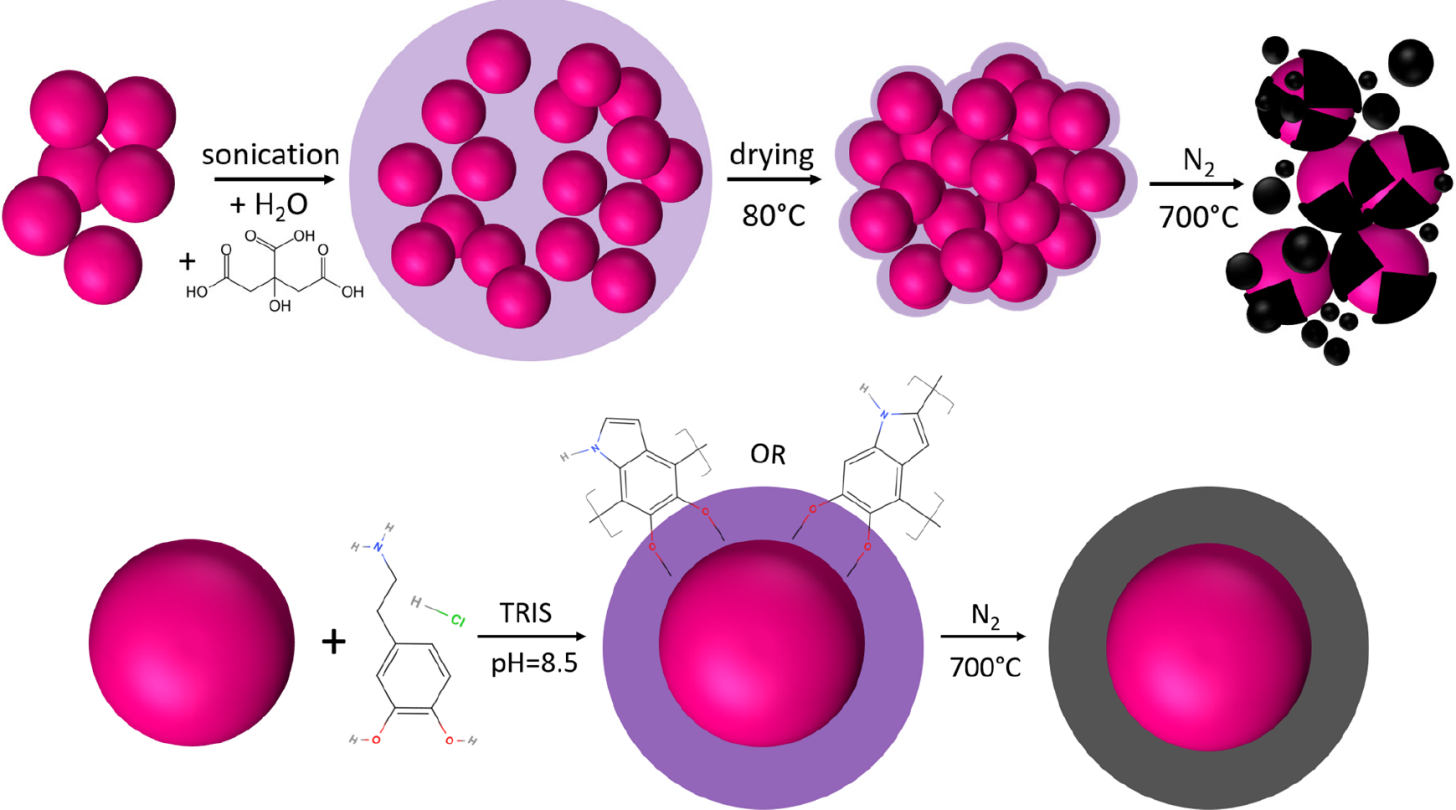
Schematic view of carbon-coated NTP particle preparation by (top) CA and (bottom) PDA encapsulation approaches.

The resulting materials are characterized in terms of their structure, morphology and electrochemical properties. The obtained results are compared to those obtained by a conventional route based on citric acid (CA).^25−27^ In the latter procedure, NTP particles are simply dispersed in CA solution and then pyrolyzed in inert gas atmosphere.^6^ The main differences between the two carbon encapsulation approaches are schematically compared in Figure 1. The results show that different carbonization strategies yield very different final carbon loadings and their distribution in the samples. The PDA route is shown to yield much more uniform encapsulation of particles at lower carbon contents than the conventional CA route. In addition, an optimized DA polymerization procedure for coating of NTP particles is designed that results in superior stability and charge capacity retention during galvanostatic charge/discharge cycling in standard flooded and naturally aerated electrochemical cells. We believe these results to be applicable not only to NTP but also to other particulate battery electrode materials stable under typical carbon pyrolysis conditions and requiring conformal electron transporting particle coatings.

## Experimental Section

### Active Material Synthesis

NaTi_2_(PO_4_)_3_ particles used in this work were synthesized via a conventional coprecipitation method. In a typical synthesis, 7.282 g of concentrated H_3_PO_4_ (Reachem, 85 wt %) was poured into a beaker and diluted with a small amount of water to reduce viscosity. 1.317 g of Na_2_CO_3_ powder was added into H_3_PO_4_ solution and left to react under continuous stirring. Then, 14.112 g of Ti(OC_4_H_9_)_4_ (Acros Organics, ≥ 98%) was added into isopropanol (20 mL) to prevent contact with air and agitated. Then it was immediately poured into the Na_2_CO_3_ and H_3_PO_4_ containing solution. The mixture was kept at room temperature to react completely for 30 min under constant stirring. In order to obtain the desired precursor, water and isopropanol were evaporated from the mixture on a hot plate. The resulting powder was ground and calcined in a muffle furnace at 700 °C in an air atmosphere for 8 h. The obtained particles were reground and additionally ball-milled for 1 h at 900 rpm in isopropanol with subsequent drying at 80 °C.

### Active Material Carbon Coating

The synthesized particles were carbon coated by homogeneously mixing NTP powder and citric acid in distilled water (50 mL). The amount of citric acid was set to be 10, 25, 40, 100, and 150 wt % of active material weight (denoted by sample codes NTP@CA1, NTP@CA2, NTP@CA3, NTP@CA4, and NTP@CA5, respectively). The resulting mixture was heated at 60 °C under magnetic stirring, and subsequently dried at 80 °C for water elimination. The obtained white powder was reground and pyrolyzed at 700 °C for 2 h in a tube furnace under constant N_2_ gas flow. The resulting powder was additionally ball-milled for 1 h at 350 rpm in isopropanol and subsequently dried at 80 °C in a drying oven.

The polydopamine coating of the NTP particles was accomplished through an *in situ* polymerization process. First, a series of tris-HCl buffers were prepared in order to keep the ratio between DA and tris constant. 0.9 g of as-prepared NTP was dispersed in tris-HCl (pH 8.5, 30 mL) buffer solution. Then, dopamine hydrochloride was added in order to achieve homogeneous polymerization on the particle surface. The amount of dopamine hydrochloride was chosen to be 5, 10, 30, 60, 100, 150, and 15 wt % of active material weight (denoted as samples @PDA1, @PDA2, @PDA3, @PDA4, @PDA5, @PDA6, and @PDA7, respectively). The mixture was dispersed by vigorous stirring at room temperature between 16 and 70 h, and then centrifuged at 7000 rpm for 10 min in order to isolate the precipitate. The collected precipitate was washed with deionized water three times and dried in a drying oven at 70 °C. The obtained brownish powder was reground and pyrolyzed at 700 °C for 2 h in a tube furnace under constant N_2_ gas flow. The resulting particles were ball-milled for 1 h at 350 rpm in isopropanol and subsequently dried at 70 °C. Table 1 summarizes the sample nomenclature and preparation conditions.

**Table 1:** Summarized Preparation Conditions and Nomenclature of the Samples in This Study

Sample code	@CA1	@CA2	@CA3	@CA4	@CA5	@PDA1	@PDA2	@PDA3	@PDA4	@PDA5	@PDA4#2	@PDA5#2	@PDA6	@PDA7
Carbon precursor, wt %	10	25	40	100	150	5	10	30	60	100	60	100	150	15
Polimerizationreaction duration, h	-	-	-	-	-	16	16	16	16	16	70	70	70	16

### Electrode Preparation

The electrode slurry was prepared by mixing 70 wt % active material, 20 wt % carbon black (CB) (Super-P, TIMCAL), and 10 wt % polyvinylidene fluoride (PVDF) (HSV1800, Kynar) in *N*-methyl-2-pyrrolidone (NMP) (Sigma-Aldrich, 99.5%). Dry components were premixed in a high-energy ball-mill for 1 h at 175 rpm. The slurry was then homogenized for 2 h at 350 rpm and subsequently casted as a film. After drying in vacuum for 3 h at 120 °C the resulting electrode film was punch-cut into 14 mm diameter disks and transferred onto 316L stainless steel mesh (#325) by hydraulic pressing.

### Materials Characterization

Infrared spectra were recorded by FT-IR spectrometer (Frontier, PerkinElmer), equipped with an attenuated total reflectance (ATR) accessory. The measurements were performed in the 4000–450 cm^–1^ range with 4 cm^–1^ resolution. The thermogravimetric determination of the surface carbon and nitrogen content was carried out on a PerkinElmer STA6000 analyzer in the range of 30 to 700 °C and a heating rate of 10 °C min^–1^ in flowing air atmosphere (20 mL min^–1^). The carbon and nitrogen contents were independently determined by organic elemental analyzer (Thermo Scientific Flash 200). The surface area was measured by a Brunauer–Emmett–Teller (BET) analyzer (Anton Paar). Morphological characterization was carried out using G2 F20 X-TWIN FEI transmission electron microscope (TEM). ImageJ software^28^ was used for carbon layer thickness determination. X-ray photoelectron spectroscopy (XPS) analyses were performed using monochromatic Al–Kα radiation (*h*ν = 1486.7 eV) at 225 W X-ray gun power at 10^–8^ Torr pressure and room temperature. The powder X-ray diffraction (XRD) patterns were recorded on a X-ray diffractometer (Bruker D2 Phaser) within the range 10° < 2θ < 60° using Ni-filtered Cu K_α_ radiation. The scanning speed and step width were 1° min^–1^ and 0.01°, respectively.

### Electrochemical Characterization

Electrochemical performance of the electrodes was evaluated by galvanostatic charge/discharge cycling (GCD) in a bottom-mount beaker-type cell designed for flat samples in Na_2_SO_4_ (aq.) (10 mL, 1 M) electrolyte solution. The working NTP and graphite rod counter electrodes were placed in separated compartments connected by 1 M NaNO_3_ agarose salt bridge. Hg/Hg_2_SO_4_/K_2_SO_4_ (aq. sat) (MSE) was used as a reference electrode. The electrolytes were naturally aerated during all experiments. The GCD cycling was carried out on a Neware CT-4008 battery cycler.

## Results and Discussion

### Structural and Morphological Characterization

**Figure 2:**
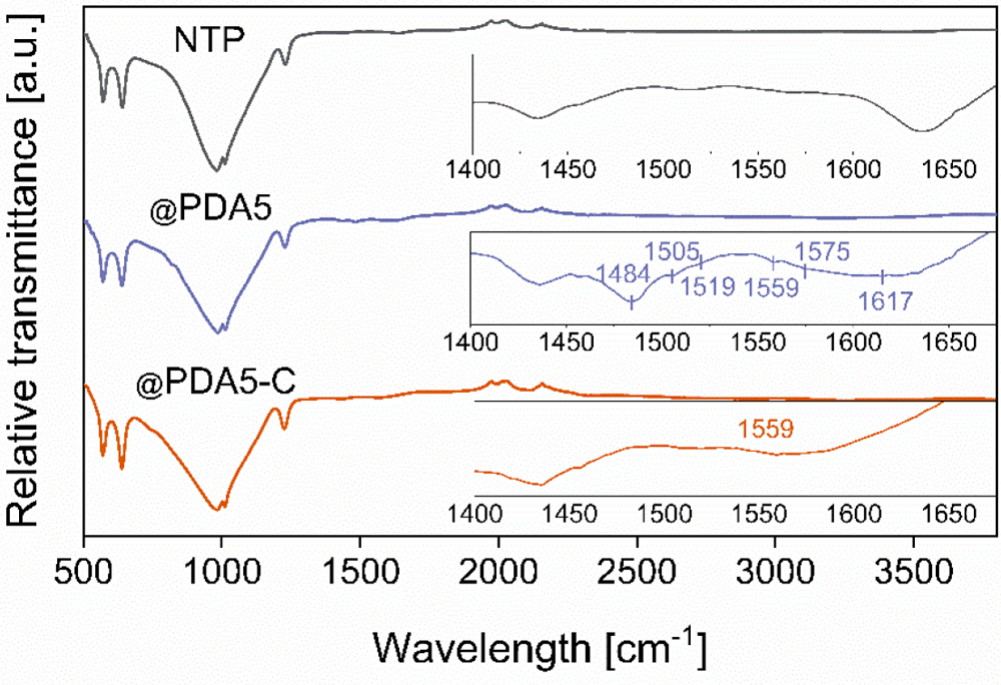
FT-IR spectra of pure NTP, NTP@PDA5, and NTP@PDA5-C samples.

The process of successful *in situ* polymerization of DA on the particle surface was first characterized by FTIR spectroscopy (Figure 2). The observed bands in the 550–1250 cm^–1^ spectral range correspond to characteristic symmetrical (570 and 640 cm^–1^) and asymmetrical (981, 1011, and 1227 cm^–1^) stretching vibrations of PO_4_ units.^6,29,30^ The characteristic vibrational bands of PDA lie in the shorter wavelength range. The features at 1505 and 1617 cm^–1^ can be attributed to resonance vibrations of the aromatic C=C bonds typical for indole rings.^31^ The bands at 1519 and 1575 cm^–1^ are indicative of scissoring and stretching N–H vibrations, respectively.^32,33^ The most prominent bands at 1486 and 1559 cm^–1^ are due to C–H and C–C/C–N bending, respectively.^34,35^ The FTIR spectrum of a pyrolyzed @PDA5-C sample in Figure 2 shows only one remaining band at 1559 cm^–1^, confirming that all PDA was successfully pyrolyzed.

**Figure 3:**
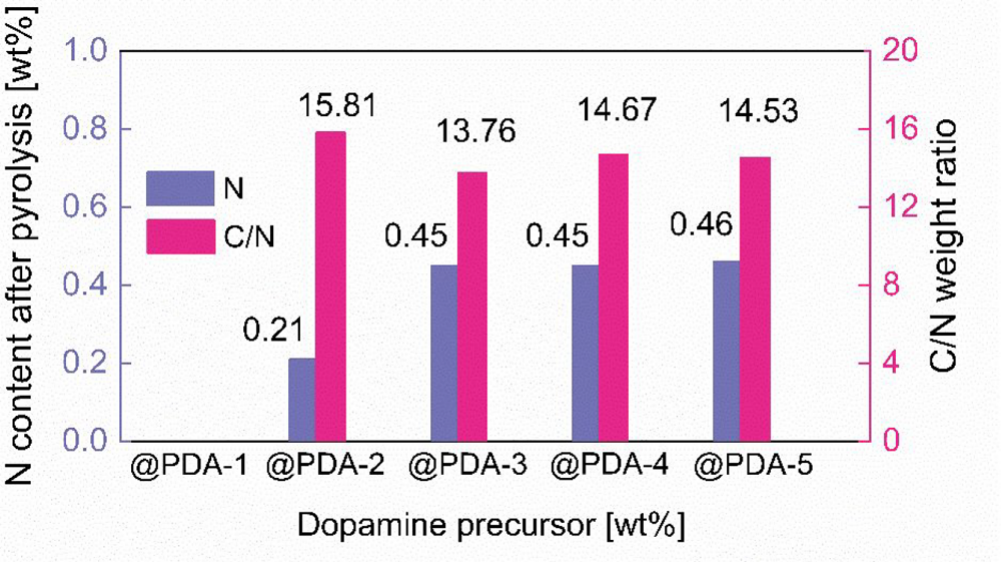
Nitrogen content and calculated C/N weight ratio for NTP@PDA-C sample series as determined by combustion elemental analysis.

The presence of bands corresponding to C–N bonds in samples before pyrolysis also indicate that resulting carbon layers might contain an enhanced concentration of nitrogen. N-doped carbonaceous phases are known to show increased catalytic activity and electronic conductivity.^36^ In order to determine the precise nitrogen content in PDA derived composites, combustion elemental analysis was carried out. The results summarized in Figure 3, indeed indicate the presence of a N-containing carbonaceous phase in these samples. The calculated ratio between carbon and nitrogen (C/N ratio) remains almost constant at ∼15 throughout the sample series, indicating a compositional consistency of PDA derived carbon coatings. It is important to note that @PDA1 sample coated with 5 wt % PDA was very close to the N content detection limit and might have a higher probability of error. These results support the view that PDA approach yields N-containing carbon coatings, whereas CA derived carbon without additional N-containing precursors (e.g., urea) is N-free.

**Figure 4:**
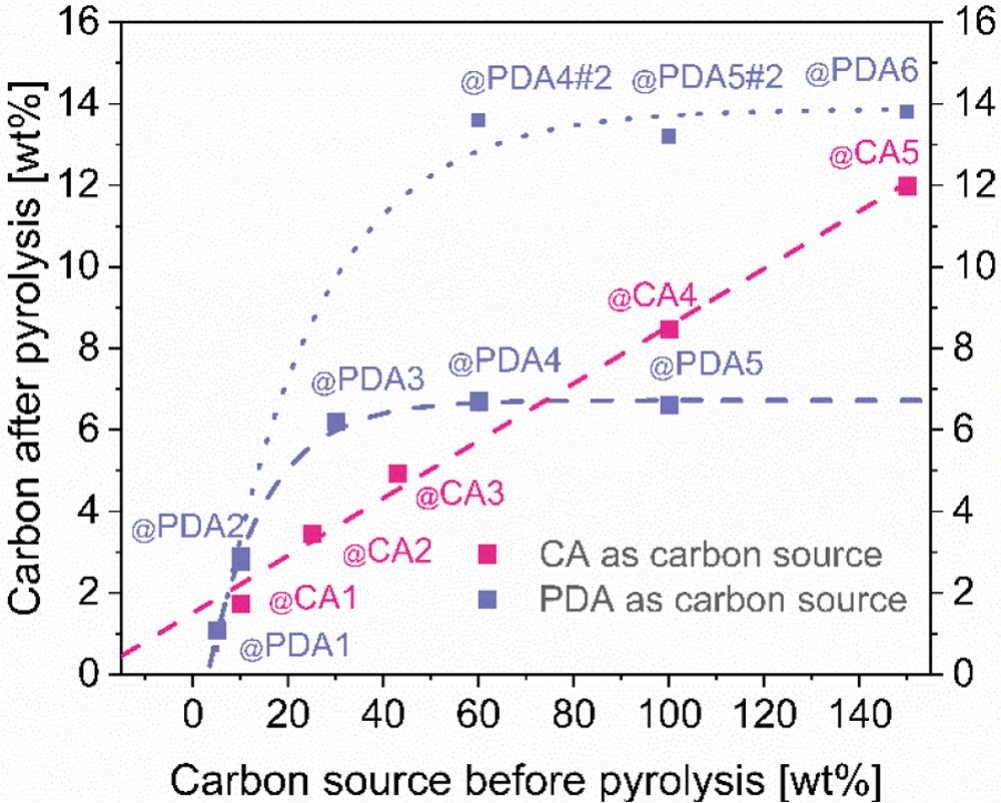
TGA determined carbon content in NTP@CA-C and NTP@PDA-C series.

Thermogravimetric analysis (TGA) allows us to quantitatively determine carbon content. Figure 4 shows that PDA route yields a very different carbon loading from CA route. The conventional CA approach results in carbon content which depends roughly linearly on the initial precursor concentration before pyrolysis. However, in the case of PDA, a linear dependence is observed only at low precursor concentration which is followed by a plateau starting at ∼20% initial precursor concentration. This means that in this stage, the final carbon loading of ∼6.5 wt % is achieved irrespective of DA concentration before polymerization. This result suggests that either some sort self-limiting surface or time-limited polymerization reaction is taking place. Another set of identical samples were prepared with a prolonged polymerization time (70 h). Figure 4 shows that, although the general tendency remains similar to a linear dependence observed at low DA concentrations, a plateau is seen at higher carbon loadings. A more than 4-fold increase in reaction time results in approximately doubling of the final carbon loading. These findings suggest that, in the case of excess DA, the reaction is slow and limited only by the polymerization time. In principle, because the unreacted monomer could be easily eliminated by repeated washing, a careful optimization of this approach enables to obtain highly controllable and reproducible carbon loading in NTP and similar materials.

**Figure 5:**
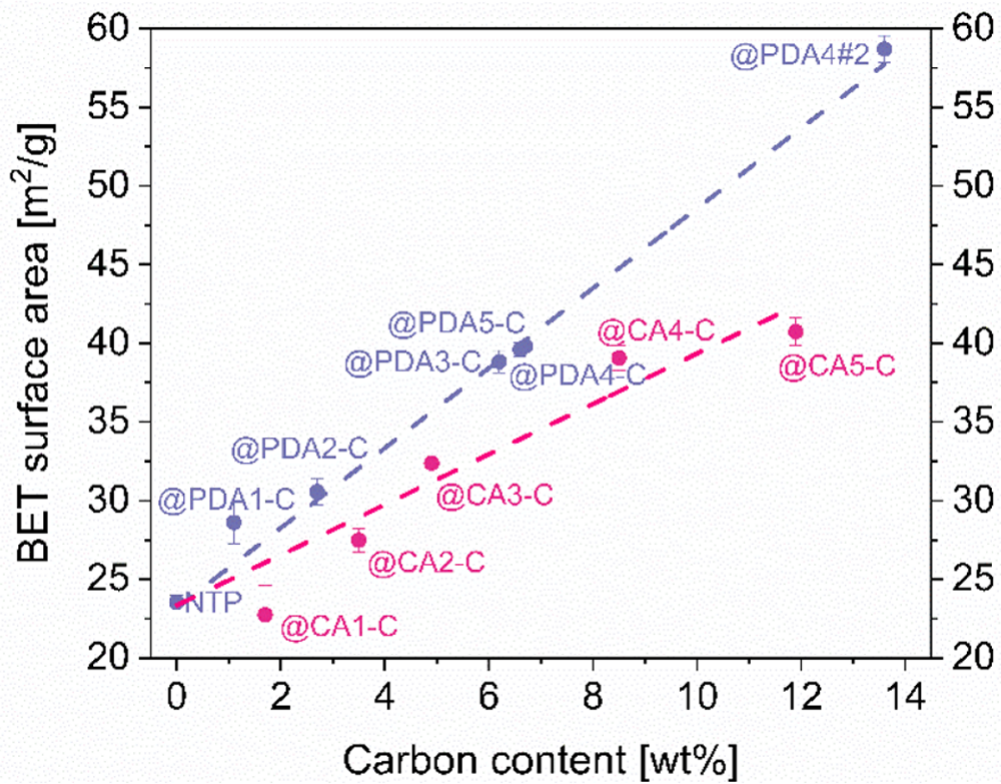
BET-determined specific surface area dependence on the final carbon loading for the NTP@PDA-C and NTP@PDA-C series of samples.

The results of BET surface area analysis of NTP@C composites are presented in Figure 5. They indicate a clear linear dependence between the measured specific surface area and the carbon content. Although the overall trends for different carbonization approaches is similar, slight but consistent differences could be also observed. CA coated series show more scattered and less consistent values, whereas PDA series exhibit very consistent dependence pointing to the superior reproducibility of this approach. The measured specific surface areas are also systematically higher in samples obtained by the PDA method.

**Figure 6:**
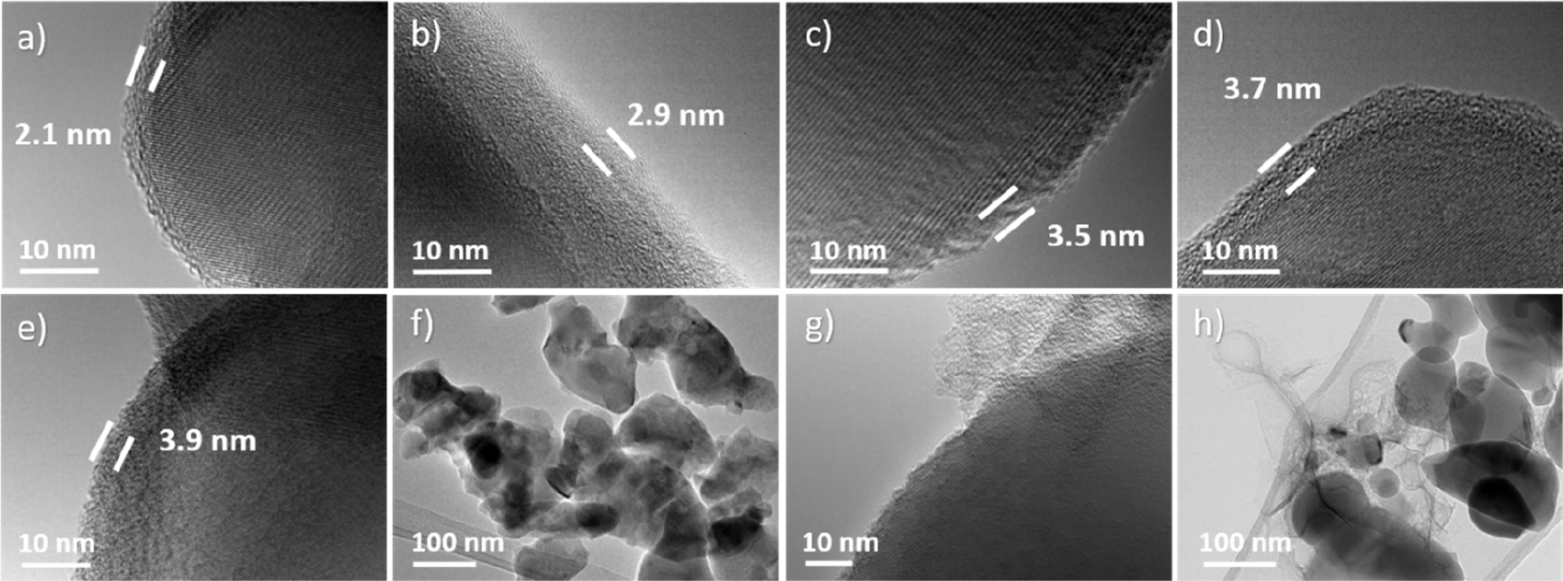
TEM images of (a) @PDA1-C, (b) @PDA2-C, (c) @PDA3-C, (d) @PDA4-C, (e, f) @PDA5-C, and (g, h) @CA5-C particles at different magnification.

In addition, TEM imaging was carried out in order to investigate the nanoscale morphology and uniformity of carbon layers on NTP particle surfaces. Figure 6a–e shows TEM images of the @PDA-C series which indicate a highly crystalline nature of the NTP, and a 2.1 to 3.9 nm thick top layer of amorphous carbon. PDA pyrolysis produces a thin but uniform and continuous layer resulting in an NTP@C core–shell structure. Further TEM examination does not indicate any separate carbon particles or intraparticular structures (Figure 6f). Instead, CA-derived carbon shows significantly less regularity and integrity on the active material particle surface and produces a coating with the thickness of ∼1.7 nm to ∼28 nm (Figure 6g). In addition, the image at lower magnification shows that chunks of carbon resulting from CA pyrolysis are present between particles in this sample (Figure 6h).

**Figure 7:**
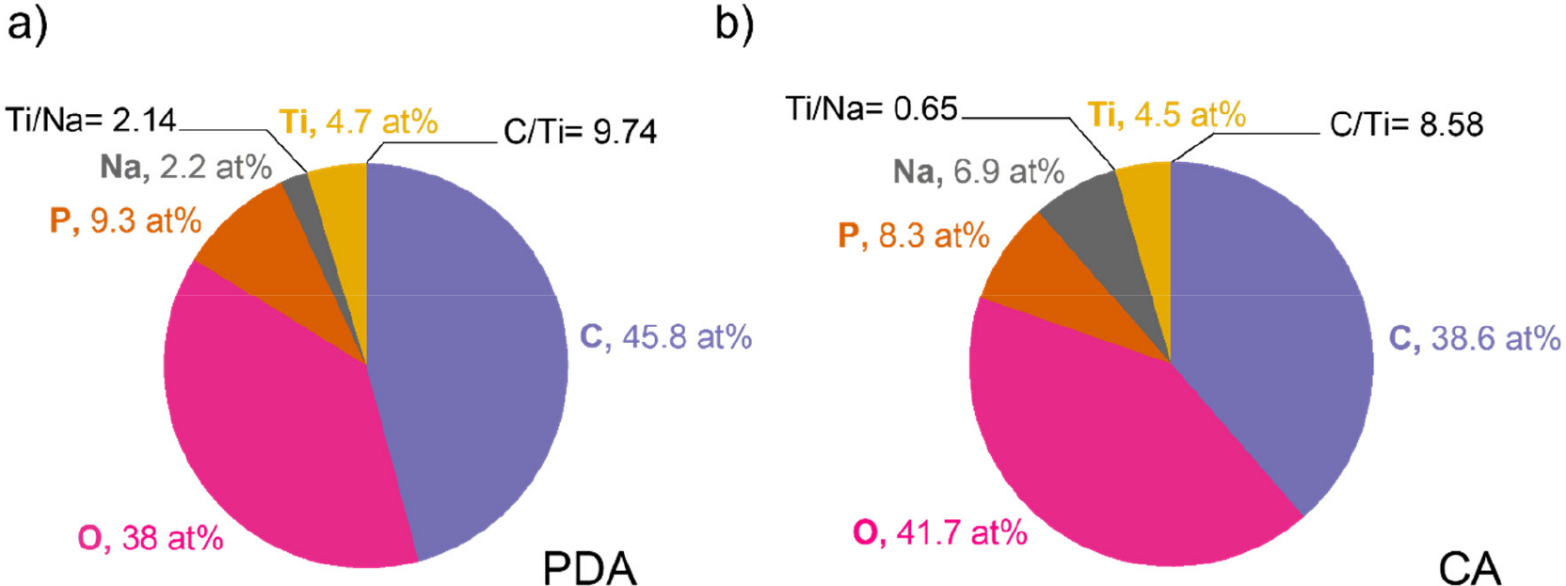
XPS analyzed surface atomic composition of (a) NTP@PDA-C and (b) NTP@CA-C samples.

In order to understand the chemical differences at the surface level of obtained materials, XPS quantitative analysis was also performed on two representative samples from both CA and PDA series having similar carbon content of 4.92% (@CA3-C) and 4.31% (@PDA7-C). Figure 7 shows the summary of surface atomic compositions as obtained by XPS. Although NTP@PDA-C samples show systematically lower carbon loading than NTP@CA-C samples, their surface contains slightly higher C/Ti ratio. This result could be an indication of a more uniform PDA-derived carbon layer on NTP particles. Another interesting result revealed by XPS analysis is that the ratios of other elements in NTP@PDA-C samples are much closer to the stoichiometrically expected ones. This indicates that the surface of PDA coated samples is likely to be more crystalline and closer to the nominal NTP composition.

**Figure 8:**
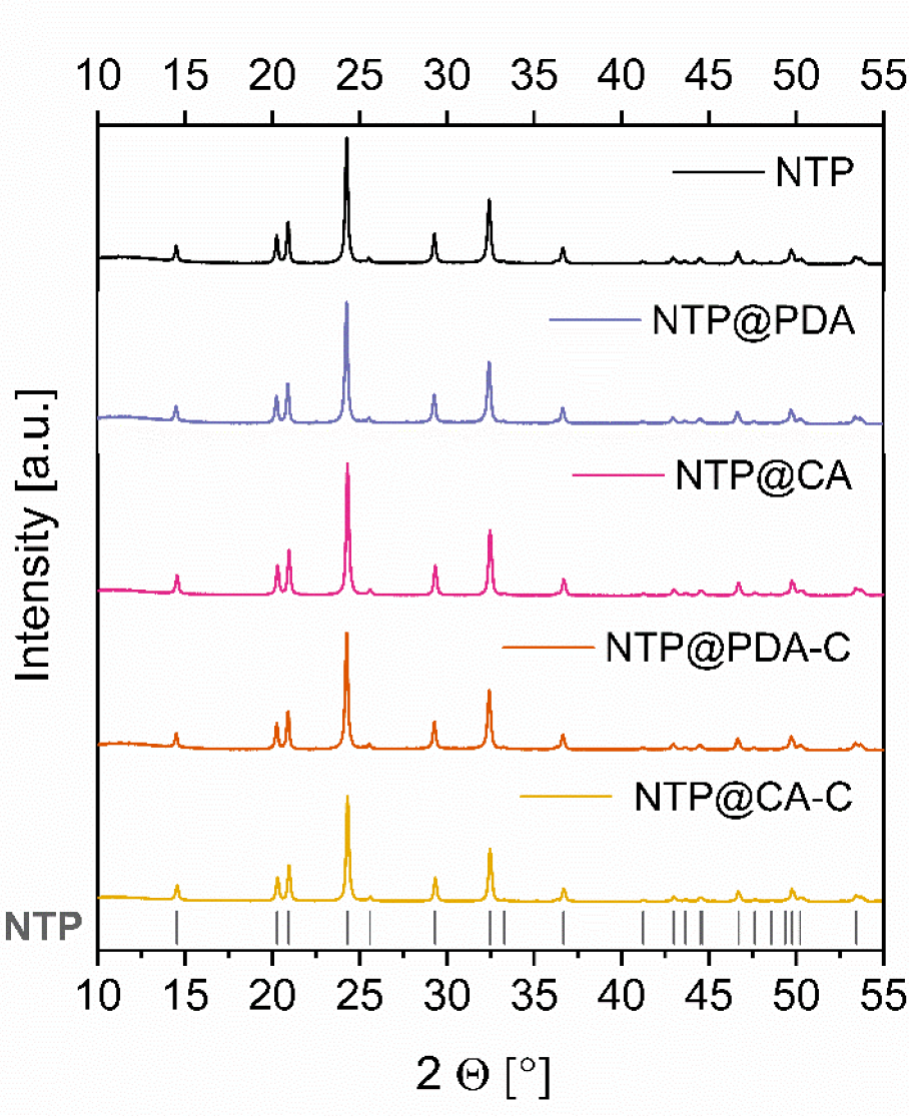
Powder XRD patterns of pure NTP and high-carbon content samples obtained by different coating strategies.

Finally, the structure of samples was characterized by powder XRD. The patterns of pure NTP, @PDA5, @CA5, @PDA5-C, and @CA5-C shown in Figure 8 indicate that neither the polymer coating nor pyrolysis procedure had any effect on the bulk NTP phase composition or crystallinity. The diffraction peaks of all three samples are very sharp and in good agreement with the standard PDF card (PDF No. 96-153-0650). The results confirm the presence of a highly crystalline phase with an ordered NASICON-type structure and a space group of R3̅c (No. 167). The recorded patterns also indicate that in even very high carbon content samples such as @PDA5, @CA5, @PDA5-C, and @CA5-C, no additional carbon peaks are present. This supports our view that all carbon is in the amorphous state.

### Electrochemical Characterization

**Figure 9:**
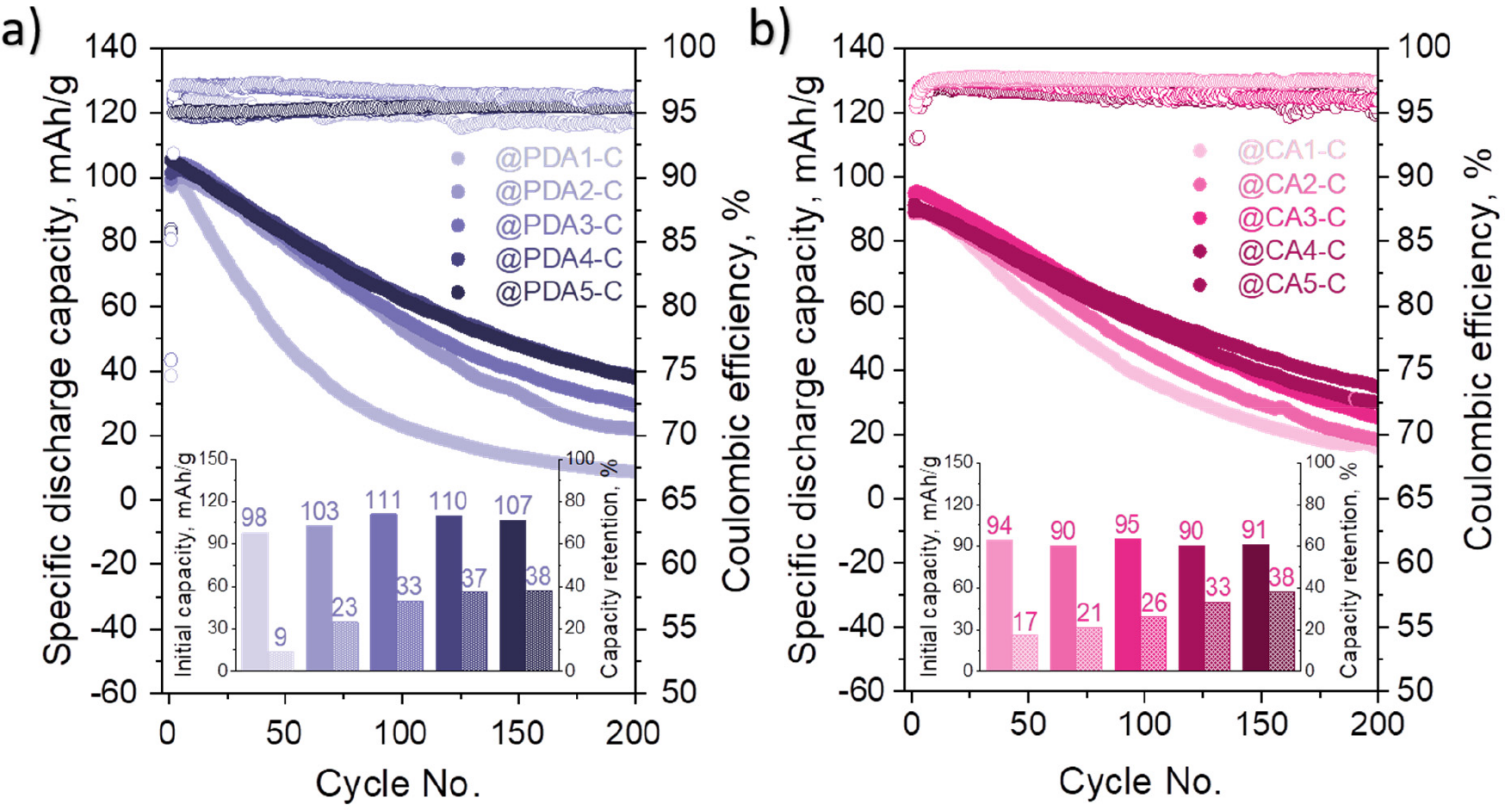
Galvanostatic charge/discharge cycling performance of (a) NTP@PDA-C and (b) NTP@CA-C electrodes in naturally aerated 1 M Na2SO4 (aq.) at a 1*C* rate.

The specific discharge capacity and capacity retention of NTP anodes prepared from coated particles were investigated by means of GCD cycling with the cutoff voltages of −0.95 V and −1.35 V (vs MSE) at a 1*C* rate (1*C* = 0.133 A g^–1^ based on the theoretical NTP capacity of 0.133 Ah g^–1^). Figure 9 represents the results of GCD cycling for NTP@CA-C and NTP@PDA-C sample series. The obtained electrochemical data on the initial charge capacities and cycling stability demonstrate the systematic superiority of NTP@PDA-C samples versus NTP@CA-C. PDA-derived composites show initial discharge capacities in the 98–111 mAh/g range, while CA-derived ones delivered around 90–95 mAh/g. Higher initial capacities could be attributed to enhanced electronic conductivity due to a more even and homogeneous N-rich carbon layer. In the case of NTP@PDA-C, there is a distinguishable increase in capacity values from NTP@PDA1-C to NTP@PDA3-C, revealing that higher carbon loadings are beneficial for PDA coated NTP electrodes. On the other hand, CA-coated series show that in this case carbon amount has little influence on the initial capacity of NTP. The difference in capacity retention after 200 cycles between the series is much more pronounced (Figure 9 (inset)). Overall, capacity retention increases with carbon content in both sample series. The highest retention of 38% after 200 cycles is obtained for the highest carbon loading samples in both cases. However, the actual carbon content in these samples is different by a factor of almost two: 11.9 and 6.7 wt % for NTP@PDA5-C and NTP@CA5-C samples, respectively. Oxygen induced self-discharge is well-known to be the leading cause for the locally increasing pH, which leads to NTP degradation and dissolution into the electrolyte.^3,37^ The results show that a more continuous and uniform core–shell NTP@C structure in PDA-coated samples is a much more effective protection from O_2_ attack, which is always present in naturally aerated electrolytes. The observed increase in cycling stability with higher carbon content could be explained by the formation and growth of an aqueous interphasial layer similar to the solid-electrolyte interphase in nonaqueous electrolytes.^3,38^ This layer composed from insoluble Ti-rich NTP degradation products is known to form at the aqueous/NTP interface and is able to locally slow down but not completely prevent the material from degradation. It has also been shown that the growth of an aqueous interphasal layer during cycling is much more pronounced in higher-carbon-content, more-porous structures.^6^

## Conclusions

In this work, two series of NaTi_2_(PO_4_)_3_ - carbon composite structures were successfully prepared by two different approaches. The first is based on the widely used postsynthesis pyrolytic treatment of a carbohydrate such as citric acid. The second, on the selective adsorption of precursors such as dopamine on the active material particle surface which can then be selectively polymerized. The pyrolysis of such polymeric layers results in hierarchical core–shell nanostructures. The results show that both synthesis routes yield electrochemically active materials with good initial capacity and decent capacity retention in naturally aerated cells. However, the polydopamine approach allows for a superior carbon loading control which can be adjusted by polymerization reaction time. The subsequent pyrolysis of a polydopamine coating results in a more continuous and uniform carbon layer yielding NaTi_2_(PO_4_)_3_–carbon core–shell structures. The successful formation, homogeneity, and uniformity of polydopamine derived carbon layers is supported by FTIR, TEM and XPS analyses, whereas combustion elemental analysis also shows the presence of N-doping. Powder X-ray diffraction results show that neither citric acid nor polydopamine pyrolysis have any effect on the NaTi_2_(PO_4_)_3_ particle crystallinity or phase composition. Finally, the galvanostatic charge/discharge cycling results show similar initial capacities and charge capacity retention. However, this is achieved at only half of the carbon loading in polydopamine derived samples versus the citric acid derived ones. The overall results indicate that the careful control and engineering of NaTi_2_(PO_4_)_3_–carbon particle nanomorphology allows us to prepare materials with superior properties and stability for various electrochemical applications such as aqueous batteries and deionization cells.

## Abbreviations

NASICON: sodium super ionic conductor
NTP: NaTi_2_(PO_4_)_3_
SHE: standard hydrogen electrode
DA: dopamine
PDA: polydopamine
CA: citric acid
CB: carbon black
PVDF: polyvinylidene fluoride
NMP: *N*-methyl-2-pyrrolidone
FT-IR: Fourier transform infrared
ATR: attenuated total reflectance
BET: Brunauer–Emmett–Teller
TEM: transmission electron microscope
XPS: X-ray photoelectron spectroscopy
XRD: powder X-ray diffraction
GCD: galvanostatic charge/discharge
MSE: mercury sulfate electrode
TGA: thermogravimetric analysis
